# An artificial neuromuscular junction for enhanced reflexes and oculomotor dynamics based on a ferroelectric CuInP_2_S_6_/GaN HEMT

**DOI:** 10.1126/sciadv.adh9889

**Published:** 2023-09-22

**Authors:** Minseong Park, Jeong Yong Yang, Min Jae Yeom, Byungjoon Bae, Yongmin Baek, Geonwook Yoo, Kyusang Lee

**Affiliations:** ^1^ Department of Electrical and Computer Engineering, University of Virginia, Charlottesville, VA 22904, USA.; ^2^ School of Electronic Engineering, Soongsil University, Seoul 06938, South Korea.; ^3^ Department of Intelligent Semiconductors, Soongsil University, Seoul 06978, South Korea.; ^4^ Department of Material Science and Engineering, University of Virginia, Charlottesville, VA 22904, USA.

## Abstract

A neuromuscular junction (NMJ) is a particularized synapse that activates muscle fibers for macro-motions, requiring more energy than computation. Emulating the NMJ is thus challenging owing to the need for both synaptic plasticity and high driving power to trigger motions. Here, we present an artificial NMJ using CuInP_2_S_6_ (CIPS) as a gate dielectric integrated with an AlGaN/GaN-based high-electron mobility transistor (HEMT). The ferroelectricity of the CIPS is coupled with the two-dimensional electron gas channel in the HEMT, providing a wide programmable current range of 6 picoampere per millimeter to 5 milliampere per millimeter. The large output current window of the CIPS/GaN ferroelectric HEMT (FeHEMT) allows for amplifier-less actuation, emulating the biological NMJ functions of actuation and synaptic plasticity. We also demonstrate the emulation of biological oculomotor dynamics, including in situ object tracking and enhanced stimulus responses, using the fabricated artificial NMJ. We believe that the CIPS/GaN FeHEMT offers a promising pathway for bioinspired robotics and neuromorphic vision.

## Teaser

A 2D ferroelectric HEMT enables amplifier-less and programmable actuation.

## INTRODUCTION

The somatosensory system in biological organisms is responsible for detecting and responding to various external stimuli, including vision, sound, odor, pressure, and temperature (*1*–*3*). To achieve these reactions, external stimuli detected by the afferent nerve (sensory neurons) are first transferred to the central nervous system (CNS) (*4*). The CNS then generates an action potential for the efferent nerve (motor nerve) that actuates the target muscle through the neuromuscular junction (NMJ). The NMJ is a unique and essential synaptic connection between the efferent nerve and muscle fibers that triggers motion via the transmission of action potentials through it (*5*). Consequently, the stimulated muscle fibers contract and relax, becoming capable of triggering macro-motions. Macro-motions generally require substantially higher energy than that required for computation. Therefore, it has been challenging to emulate NMJs to fulfill both synaptic plasticity and the capability to drive large amounts of energy (*6*–*9*).

To address these challenges, here we demonstrate synaptic transistors by heterogeneously integrating a CuInP_2_S_6_ (CIPS) ferroelectric membrane as a gate dielectric material with an AlGaN/GaN high-electron mobility transistor (HEMT). The programmable transconductance of ferroelectric transistors allows for artificial synaptic plasticity, which is attributed to the polarization of the ferroelectric gate dielectric layer. Among many ferroelectric materials, including Zr-doped Hf_1−*x*_Zr*_x_*O (*10*), PbZr_0.52_Ti_0.48_O_3_ (*11*), BaTiO_3_ (*12*), α-In_2_Se_3_ (*13*), and BiFeO_3_ (*14*), CIPS is a unique two-dimensional (2D) van der Waals (vdW) material with a relatively wide bandgap and out-of-plane ferroelectricity that offers high integrability, flexibility, and responsivity to electrical signals (*15*–*20*). The out-of-plane ferroelectricity in the CIPS is attributed to the ionic dynamics of the Cu and In cations, which are vertically displaced in the sulfur framework (*21*).

We use a CIPS/GaN ferroelectric HEMT (FeHEMT) to achieve high-power driving capability. GaN-based HEMTs are widely used in radio frequency (RF) and power electronics applications because of their high output current and fast switching capability by using an AlGaN/GaN heterostructure, which forms a 2D electron gas (2DEG) transport channel (*22*). Thus, it offers a high-electron saturation velocity, a high breakdown electrical field, and a high-electron mobility suitable for high-power and high-frequency applications (*23*, *24*).

The fabricated CIPS/GaN FeHEMT exhibited hysteresis in the current-voltage (*I*-*V*) characteristics that emulate biological short/long-term plasticity (STP/LTP) (*25*). The feasibility of the CIPS/GaN FeHEMT as an artificial synapse was experimentally verified by characterizing its programmability, retention time, and endurance. In addition, we demonstrate enhanced reflexes by accelerating the spike timing of the ferroelectric switching in CIPS/GaN FeHEMT, analogous to the enhanced response to the stimulus in biological systems. The enhanced reflexes were achieved by connecting a CIPS/GaN FeHEMT with a complementary metal-oxide semiconductor (CMOS)–based integrate fire unit (IFU) that accumulates the input spikes and fires the output spike when the accumulated spike exceeds a threshold. Last, we used a CIPS/GaN FeHEMT for direct synaptic power transmission to the microelectromechanical system (MEMS)–based actuators without amplifier circuits, mimicking the oculomotor NMJ that triggers muscular motion of the eyeball for in situ object tracking. We believe that the proposed device can potentially play a key role in the implementation of artificial NMJ for robotic and artificial muscle systems.

## RESULTS

**Fig. 1.**
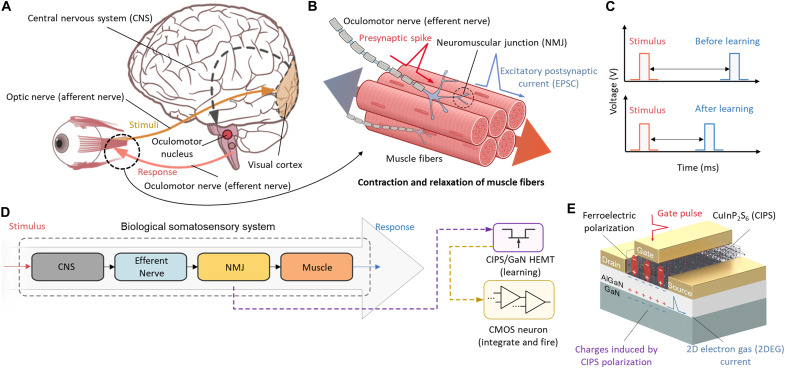
Schematic illustration of mimicking NMJ. (**A**) Schematic illustration of oculomotor system. The stimulus transmitted through the optic nerve propagates toward the target muscle via the CNS, oculomotor nucleus/nerve, and NMJ. (**B**) Biological NMJ. The NMJ connects the terminal of the oculomotor nerve with muscle fibers. (**C**) Emulation of the NMJ for fast stimulus enhancement. The fast response is emulated by the programmable CIPS/GaN FeHEMT and IFU. (**D**) Emulation of NMJ dynamics. The CNS generates an action potential on the efferent nerve that actuates the target muscle through the NMJ. The enhanced CIPS/GaN FeHEMT with an IFU allows for enhanced reflexes. (**E**) Schematic of CIPS/GaN FeHEMT structure. The polarization of the CIPS/GaN FeHEMT mimics synaptic plasticity and generates a programmable 2DEG current that serves as an artificial EPSC.

Figure 1A shows a schematic of the biological oculomotor system. When visual stimuli via the optic nerve—categorized as an afferent nerve—trigger the CNS (*4*), the CNS generates an action potential via the oculomotor nucleus in the midbrain. The potential is then transmitted to the NMJ via the oculomotor nerve—categorized as an efferent nerve—and lastly actuates the target muscle (*26*). Figure 1B illustrates the neuromuscular dynamics driven by stimuli from the CNS. The NMJ connects the oculomotor nerve to the muscle fibers, generating an excitatory postsynaptic current (EPSC) that triggers contraction and relaxation of the muscle fibers (*27*). Repeated stimuli reinforce synaptic connectivity for signal transmission between NMJs and muscle fibers in the somatosensory system, resulting in enhanced reflexes (*6*). Trained athletes’ quick reactions—such as sprint starts, swim starts, and dribbling—are good examples of enhanced conscious responses. In this work, we demonstrated the enhancement using artificial synaptic plasticity of the ferroelectric field-effect transistor through an enhancement process (Fig. 1C).

We used a ferroelectric CIPS membrane integrated into a GaN HEMT as a gate dielectric layer (Fig. 1D) to emulate neuromuscular synaptic plasticity and motion driving capability. The ferroelectric polarization of CIPS enables the GaN HEMT to be programmable, which is a critical function of biological STP and LTP. We also emulated the enhanced response by integrating artificial synapses based on the CIPS/GaN FeHEMT as an NMJ with an IFU that accumulated the incoming pulse train and generated a single firing spike (Fig. 1E).

**Fig. 2.**
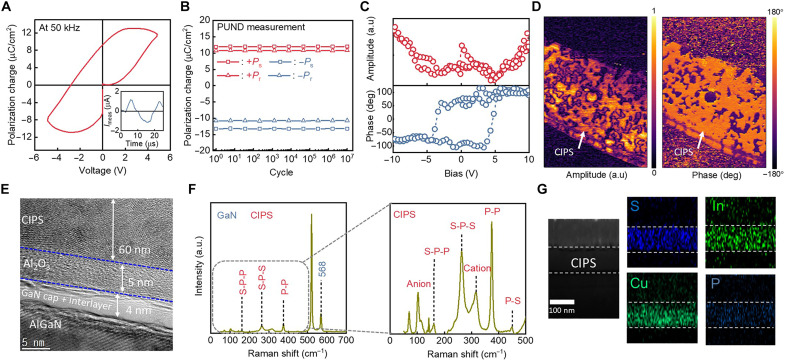
Material analysis of CIPS/GaN FeHEMT. (**A**) *P-V* characterization of Ti/Au/CIPS/Ti/Au MFM capacitor under 50-kHz triangular pulse. Inset: PUND waveform. (**B**) Polarization switching endurance of CIPS membrane. Red and blue lines denote endurance under downward and upward polarization, respectively. (**C**) PFM characterization of CIPS membrane. The hysteresis loops of both the amplitude and phase PFM verify the ferroelectricity of the CIPS membrane. (**D**) PFM imaging of CIPS membrane on metal (15 μm–by–10 μm horizontal scan). The bright region indicates the piezoelectric response of the CIPS membrane. (**E**) Cross-sectional transmission electron microscopy (TEM) image of CIPS/GaN FeHEMT. (**F**) Raman spectroscopy of CIPS/GaN FeHEMT, including GaN (left) and CIPS (right) peaks. The GaN peak is at 568 cm^−1^, and the CIPS peaks include the multiple vibration peaks (150 to 400 cm^−1^) and ionic peaks (100 and 315 cm^−1^). (**G**) Energy dispersive x-ray spectroscopy (EDS) characterization of cross-sectional CIPS/GaN FeHEMT. White dash line denotes CIPS region. Cu, In, S, and P are uniformly distributed in the CIPS region.

To analyze the ferroelectric properties of the CIPS membrane, including the polarization window, endurance, and spatial ferroelectricity, we first fabricated a two-terminal metal-ferroelectric-metal (MFM) capacitor. An optical microscopy image of the Ti/Au/CIPS/Ti/Au MFM capacitor is shown in fig. S1. Figure 2A shows the bipolar polarization-voltage (*P*-*V*) curve of the CIPS without a preset loop. The *P*-*V* curve exhibited a hysteresis loop, a remanent polarization of approximately 10 μC/cm^2^, and a coercive voltage of ±3 V driven by a 50-kHz triangular pulse. We also characterized the endurance performance using the repeated positive-up and negative-down (PUND) measurement technique (Fig. 2B). The polarization switching between saturation (*P*_s_) and remanent polarization charge (*P*_r_) persisted over 10^7^ cycles, confirming the stability and programmability of the CIPS membrane. The results of piezoelectric force microscopy (PFM) measurements are shown in Fig. 2 (C and D). The hysteresis loops in both the amplitude and phase of the PFM measurements indicated ferroelectricity in the CIPS membrane (Fig. 2C). The spatially resolved amplitude and the phase of the piezoelectric potential were mapped in the PFM images (Fig. 2D), confirming the piezoelectric response of the CIPS membrane.

Figure 2E displays a cross-sectional transmission electron microscopy (TEM) image of the CIPS membrane, which was mechanically exfoliated from the bulk crystal. The freestanding membrane was heterogeneously integrated onto an AlGaN/GaN HEMT epilayer passivated with Al_2_O_3_ as a gate dielectric through vdW forces. The thicknesses of the CIPS and Al_2_O_3_ layers were 60 and 5 nm, respectively. Figure 2 (F and G) shows the Raman spectra of the CIPS/GaN FeHEMT. The peak at 568 cm^−1^ indicates the GaN layer (Fig. 2F), and the peaks at 160, 263, and 375 cm^−1^ correspond to the S-P-P, S-P-S, and P-P vibrations of the CIPS membrane, respectively. The ionic responses of the CIPS membrane were also characterized, with P_2_S_6_^4−^ anion and Cu^+^ cation peaks observed at approximately 100 and 315 cm^−1^, respectively (*28*). Figure 2G shows the cross-sectional energy-dispersive x-ray spectroscopy (EDS) mapping results of the CIPS/GaN FeHEMT. The EDS mapping confirms that the CIPS layer is composed of uniformly distributed Cu, In, P, and S.

**Fig. 3.**
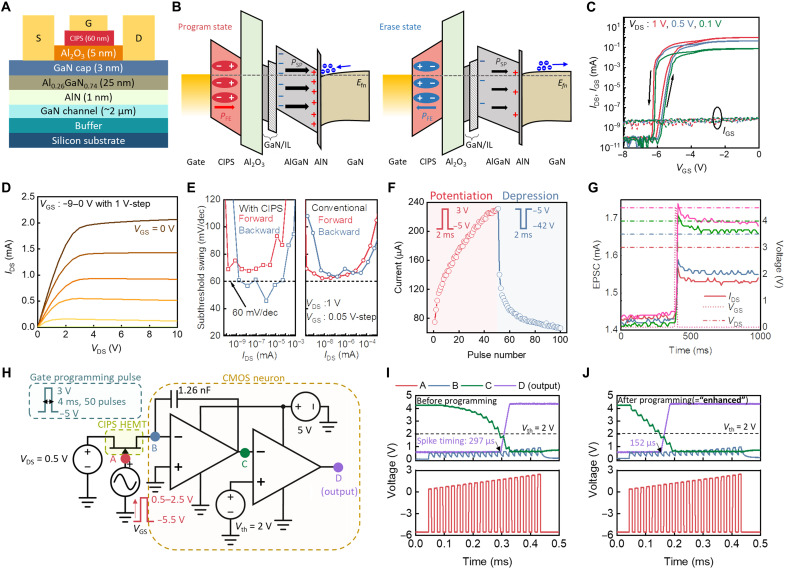
Electrical and neuromorphic characterizations of CIPS/GaN FeHEMT. (**A**) Cross-sectional device structure of CIPS/GaN HEMT. (**B**) Band diagrams of program and erase states of CIPS/GaN FeHEMT. The IL in the figure indicates an oxygen-abundant GaN interlayer between Al_2_O_3_ and AlGaN. The positive bias generates downward polarization, lowering the barrier height and increasing the transconductance of the CIPS/GaN FeHEMT. In contrast, the negative bias generates the upward polarization that increases the barrier height and decreases the transconductance of the CIPS/GaN FeHEMT. (**C**) *I-V* transfer curve of the fabricated CIPS/GaN HEMT. The double sweep switches the ferroelectric polarization of the CIPS, thus the hysteresis loop forms. (**D**) *I-V* output curve of the CIPS/GaN FeHEMT with an output current of approximately 2 mA. (**E**) Comparison of subthreshold swing (SS) between CIPS/GaN FeHEMT and GaN HEMT. With the CIPS membrane, the SS overcomes Boltzmann limitation (60 mV/dec). Emulations of biological (**F**) LTP and (**G**) STP. The positive (3 V) and negative (−42 V) gate biases (*V*_GS_) enable nonvolatile programming of the CIPS/GaN FeHEMT. (**H**) Schematic illustration of integrated CIPS/GaN FeHEMT with an IFU. Output spiking responses (**I**) before programming and (**J**) after programming (enhanced). After the enhancement process, an approximately two times faster response (output spike) was achieved (297 μs) compared to the response without the enhancement process (152 μs).

Figure 3A provides a detailed device structure of the fabricated CIPS/GaN FeHEMT, which includes a Ti/Al/Ni/Au (25/140/40/50 nm) source/drain electrode, a Ni/Au (40/50 nm) gate electrode, a CIPS/Al_2_O_3_ (60/5 nm) gate dielectric, a GaN capping layer (3 nm), Al_0.26_Ga_0.74_N (25 nm) layer, AlN (1 nm), and a GaN channel layer (~2 μm). A top-view optical microscopy image of the three-terminal synaptic transistor with the CIPS ferroelectric membrane is shown in fig. S2. Figure 3B illustrates the band diagrams for the CIPS/GaN FeHEMT under programmed and erased states, estimated by physics-based technology computer-aided design (TCAD) simulations (see fig. S3 for details). An external electric field alters the polarization state of the CIPS gate dielectric. A positive bias generates a downward polarization state that reinforces the spontaneous polarization of AlGaN and increases the electron carrier concentration in the 2DEG of the HEMT structure. In contrast, a negative bias switches the polarization state upward, decreasing the electron carrier density in the HEMT channel by counterbalancing the spontaneous polarization of AlGaN (*29*). This ferroelectric switching capability, coupled with the carrier concentration in the 2DEG through external stimuli, enables a programmable output current.

Figure 3C presents the transfer *I*-*V* characteristics of the fabricated CIPS/GaN FeHEMT during forward and backward bias sweeps. The forward sweep led to the switching of out-of-plane polarization of the CIPS/GaN FeHEMT, decreasing a threshold voltage up to 0.5 V with an ~1.5 × 10^8^ ON/OFF ratio during the backward sweep. The gate leakage current (*I*_GS_) of the CIPS/GaN FeHEMT was substantially smaller (~5 pA) than the on current (*I*_DS_, 0.95 mA). These results were consistent with the proposed TCAD model (fig. S4), corresponding to the program and erase states of the energy band diagrams, respectively (fig. S5). During a backward sweep, the CIPS polarization changed downward and enhanced the polarization in the AlGaN barrier layer. The CIPS down polarization induced the accumulation of 2DEG, allowing the transport channel to be more conductive. This results in an increase in *I*_DS_ at a fixed gate bias and thus negatively shifts the threshold voltage (*V*_th_) (programmed state in Fig. 3B). During a forward sweep, the depletion of the 2DEG resulted in a positive shift in *V*_th_ owing to the reduced current at a fixed gate bias (erase state in Fig. 3B).

Figure 3D shows the output *I*-*V* characteristics of the CIPS/GaN FeHEMT. The saturation current of the CIPS/GaN FeHEMT after polarized switching was 2 mA at gate voltage (*V*_GS_ = 0 V) and drain voltage (*V*_DS_ = 10 V), which corresponds to a current density of 200 mA/mm. As shown in Fig. 3E, the subthreshold swing (SS) was improved by using the CIPS/GaN vdW heterostructure due to the negative capacitance effect of the CIPS gate dielectric. The lower SS drives a sufficient current at low drain bias and suppresses a leakage current, reducing the energy consumption for neuronal circuitry such as spiking neural networks and reservoir computing (*30*). After polarization switching, the SS at the backward sweep overcomes the 60 mV/dec Boltzmann limitation (46 mV/dec) at room temperature, whereas the conventional AlGaN/GaN HEMT without the CIPS membrane only shows a SS of near 60 mV/dec. With a programming gate pulse of 2 ms, the retention time of the out-of-plane polarization persisted for more than 5 hours, which is sufficient for time-varying high-order neuromorphic computing applications (fig. S6) (*25*).

The CIPS/GaN FeHEMT allows the programming of synaptic weight, emulating biological LTP and STP, which can be used for neuromorphic computing applications. Figure 3F shows the transition in the analog output current with incremental potentiation and depression pulses, ranging from 80 to 240 μA over 50 consecutive pulses. The depletion mode of the CIPS/GaN FeHEMT requires a relatively large negative *V*_GS_ to effectively reset the device compared to the synaptic transistors that operate in enhancement mode. The negative voltage baseline of −5 V was used to characterize the LTP of the depletion-mode CIPS/GaN FeHEMT, which can potentially be tuned as an enhancement-mode device by using a recess gate structure. We used this analog behavior for the MNIST classification simulation to confirm the synaptic functionality of the CIPS/GaN FeHEMT (*31*), surmising a crossbar array structure of the CIPS/GaN FeHEMT for matrix multiplication in artificial neural network (ANN) architectures (fig. S7A) (*32*). The accuracy of the classification was comparable to that of the ANN software, achieving 82% classification accuracy (fig. S7, B and C). The trained ANN allowed robust classification capability for noised images, as shown in fig. S7D. Therefore, we confirmed that the CIPS/GaN FeHEMT-based crossbar can serve as a hardware accelerator that computes the ANN feedforward in a short time, whereas the conventional von Neumann architecture requires multiple accesses to the arithmetic and memory units for the computation (*33*). Moreover, the biological STP is emulated by a temporal synaptic response of CIPS/GaN FeHEMT. As shown in Fig. 3G, a higher presynaptic spike (*V*_DS_) generated a higher EPSC peak as the output current of the CIPS/GaN FeHEMT; in addition, a longer spike width (*t*_set_) resulted in a higher EPSC peak (fig. S8). The program and read gate voltages are 1-ms-width 4.5 and −1.6 V, respectively. The decay mechanism at the initial stage of the input pulse application was attributed to the spontaneous return of Cu^+^ ions that had previously migrated when the built-in electric field in CIPS was smaller than the external electric field (*16*, *34*). These transient EPSC characteristics of the CIPS/GaN FeHEMT were used to emulate the biological STP.

The combination of artificial LTP and STP enables spatiotemporal processing, which provides a pathway for emulating the enhanced reflexes. Figure 3H shows the CIPS/GaN FeHEMT connected to the IFU that accumulates incoming spikes and generates different spike timings with respect to the programmed states of the CIPS/GaN FeHEMT. The IFU includes integrating and firing building blocks. The integrating building block includes two amplifiers, a resistor, and a capacitor that accumulate the input voltage signals. The following amplifier serves as the firing building block, generating an output spike when the accumulated input signal exceeds the predefined threshold voltage (*V*_th_, 2 V). We applied repeated external stimulation (50 gate pulses), referred to as the enhancement process, to strengthen the synaptic connections toward the target muscle. Each pulse features a 50% duty cycle and a baseline of −5.5 V. The transconductance of the enhanced CIPS/GaN FeHEMT increased, generating larger spikes and triggering a faster corresponding spike from the comparator. After enhancement procedures, a smaller number of external stimulations (20 gate pulses) generated a higher EPSC that mimicked the increased connectivity between the biological muscle and the NMJ. The EPSC generated by the CIPS/GaN FeHEMT is accumulated by the IFU; then, by firing a final output spike upon exceeding *V*_th_, as shown in Fig. 3 (I and J), an approximately two times faster response (output spike) was achieved (152 μs) compared to the response without the enhancement process (297 μs).

**Fig. 4.**
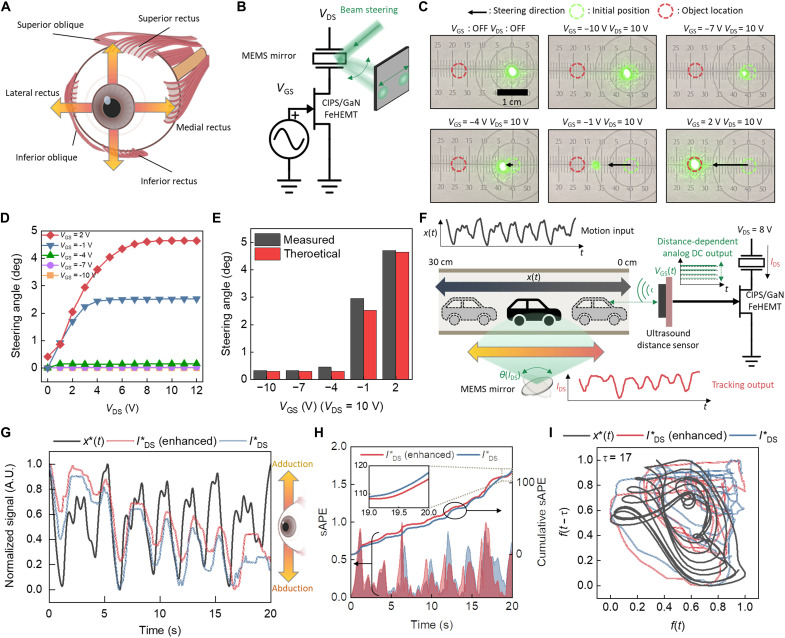
Emulation of oculomotor dynamics via artificial CIPS/GaN FeHEMT-based NMJ. (**A**) Schematic illustration of biological muscles (recti and obliques) for oculomotor dynamics. The arrows are the rotation directions, corresponding to each oculomotor muscle. (**B**) Direct amplifier-less operation of actuator via CIPS/GaN FeHEMT. The beam steering emulates the adduction and abduction motions. The two-terminal MEMS mirror is connected to the drain of the CIPS/GaN FeHEMT. (**C**) Experimental results of beam steering. The displacement of the laser beam is dependent on the steering angle of the MEMS mirror, which actuation is driven by the *I*_DS_ of the CIPS/GaN FeHEMT. (**D**) Voltage-dependent steering angles. The steering angle is proportional to *I*_DS_, modulated by *V*_GS_. (**E**) Comparison between measured and theoretical steering angles of the integrated MEMS mirror and CIPS/GaN FeHEMT. (**F**) Schematic of in situ mechanical object tracking. The tracking system additionally incorporates an ultrasound distance sensor integrated to the gate of the CIPS/GaN FeHEMT. The position of the car is captured by the ultrasound distance sensor and converted to the analog voltage that drives *I*_DS_ to steer the mirror. (**G**) Motion input and tracking output time-series data. The enhanced CIPS/GaN FeHEMT shows a higher *I*_DS_ (red line) that is more closely matched with the motion input signal for effective object tracking through beam steering. (**H**) Symmetric absolute percentage error (sAPE) plots of tracking output (*I*_DS_) with respect to motion input. The enhanced CIPS/GaN FeHEMT drives larger initial *I*_DS_, improving the cumulative sAPE of tracking. Inset: Zoom-in plot of cumulative sAPE. (**I**) Phase plots of motion input and tracking output. The enhanced system shows closer phase tracking of the motion input.

On the basis of the biological plausibility of LTP and STP, the CIPS/GaN FeHEMT exhibits programmable temporal dynamics with a high enough output current to drive external actuators. To demonstrate the feasibility of this direct actuation, we performed experiments on oculomotor dynamics using the CIPS/GaN FeHEMT-based artificial NMJ for in situ mechanical object tracking. As shown in Fig. 4A, the oculomotor dynamics are mediated by multiple extraocular muscles, including the obliques and recti. The lateral and medial recti govern the lateral rotation of the eyeballs via adduction and abduction (*35*). We integrated a MEMS mirror (S12237-03P, Hamamatsu) directly with the CIPS/GaN FeHEMT to emulate these extraocular movements (Fig. 4B). The movements were visualized by exhibiting displacements of the reflected laser beam on the MEMS mirror toward the target location, depending on the applied *V*_GS_ to the CIPS/GaN FeHEMT (Fig. 4C). We confirmed that the high driving output current of the CIPS/GaN FeHEMT was sufficient to operate the MEMS mirror without additional amplifying circuitry (Fig. 4D) by comparing the measured and calculated steering angles of the actuator (Fig. 4E).

By directly integrating a sensor into the system, the synaptic plasticity of the CIPS/GaN FeHEMT can be used to improve the in situ object-tracking functionality (Fig. 4F). We used the programmable robot car (Zumo Robot v1.2, Pololu) as a tracked object that moves back and forth based on the chaotic time series input (for more details, see Materials and Methods). The gate of the CIPS/GaN FeHEMT was connected externally with the ultrasound sensor (URM09, DFRobot) that detects the distance between the sensor and object [*x*(*t*)]. *x*(*t*) ranges from 0 to 30 cm, corresponding to the chaotic oscillation range of the robot car. The distance-dependent analog voltage from the ultrasound sensor was applied as *V*_GS_ to the CIPS/GaN FeHEMT. The output drain current (*I*_DS_) of the CIPS/GaN FeHEMT was dependent on the movement of the target object and modulated the steering angle [θ(*I*_DS_)] of the MEMS mirror for in situ object tracking.

Figure 4G confirms the in situ object tracking capability using the assembled system consisting of the ultrasound sensor, CIPS/GaN FeHEMT, and MEMS mirror. Here, we showed that the motion of the robot car stimulated the dynamics of adduction and abduction using an artificial NMJ, depending on its moving directions. The time-dependent information of the object position [*x*(*t*), black line] was converted into an electrical input signal of the CIPS/GaN FeHEMT by the ultrasound sensor. The mechanical steering [θ(*I*_DS_)] of the MEMS mirror was triggered by the ultrasound sensor and the assembled CIPS/GaN FeHEMT, corresponding to the oculomotor controls by the lateral and medial recti. We examined the CIPS/GaN FeHEMT before and after the enhancement to achieve the enhanced reflexes (blue and red line, respectively), emulating the enhancement of the synaptic connections in biological neural systems. The enhanced CIPS/GaN FeHEMT exhibited increased transconductance, resulting in enhanced *I*_DS_ for an efficient object tracking process. Figure 4H shows the error plots of *I*_DS_ with and without the enhanced process. We used a symmetric absolute error (sAPE) (*36*) to avoid undefined output for zero values

$$\mathrm{sAPE}=\frac{|x^{*}(t)-I_{\mathrm{DS}}^{*}(t)|}{[x^{*}(t)+I_{\mathrm{DS}}^{*}(t)]/2}$$ (1)

where x*(*t*) and $I_{\mathrm{DS}}^{*}$ are normalized *x*(*t*) and *I*_DS_ (between 0 and 1), respectively. As time progresses, the cumulative sAPE decreases in the enhanced state (red line), which is attributed to the programmed transconductance of the CIPS/GaN FeHEMT. We also provide a video of the in situ mechanical tracking of a moving object (robot car with multiple chaotic oscillations) using the artificial oculomotor system (movie S1). The raw data of Fig. 4G verify that the lower cumulative sAPE of the enhancement process was not merely due to the randomness of the experiment but mainly due to the enhancement process of the CIPS/GaN FeHEMT (fig. S9). Figure 4I shows the phase plots of each signal [represented as *f*(*t*)] after the baseline correction for $I_{\mathrm{DS}}^{*}$. $I_{\mathrm{DS}}^{*}$ with the enhancement process shows closer phase tracking with the motion input (movie S2).

## DISCUSSION

We have demonstrated that an artificial NMJ can generate a programmable high current by using the tunable ferroelectric coupling between the CIPS gate dielectric and 2DEG channel at the AlGaN/GaN interface. Figure S10 shows the simulated CIPS thickness-dependent hysteresis loops, revealing that the largest hysteresis with an enhanced ∆*V*_th_ is achieved with a CIPS membrane of 100 nm, while we used a 60-nm-thick CIPS membrane in this study. We believe that the programmability of CIPS/GaN FeHEMTs can be further improved by precisely controlling the CIPS thickness.

Analogous to conventional GaN HEMTs, the proposed CIPS/GaN FeHEMT also potentially attains nanosecond operation by etching the AlGaN barrier layer to minimize the surface potential and by using the thinner CIPS membrane to alleviate the voltage drops at CIPS (*22*, *37*). The direct actuation of the mechanical platform using the artificial NMJ provides a wide range of neuromorphic sensing-to-action applications, including time-of-flight ranging (*38*–*43*), in-sensor/near-sensor computing (*44*–*48*), and human-computer interaction (*49*). In this study, we achieved a normalized output current of 200 mA/mm with the CIPS/GaN FeHEMT, which is notably greater than that of recently reported synaptic transistors (for more details, see table S1) (*50*–*59*). Therefore, the CIPS/GaN FeHEMT is potentially deployable as an artificial NMJ in robotic systems to operate mechanical actuators that require a milliampere-scale driving current for macro-motion. Moreover, the 2DEG transport channel of HEMT offers a gigahertz range frequency response that can be coupled with integrated ferroelectricity for reconfigurable RF applications (*13*, *29*).

We also applied a CIPS ferroelectric membrane integrated with a GaN FeHEMT for artificial oculomotor dynamics. The high output current achieved by the AlGaN/GaN 2DEG enabled amplifier-less actuation for the adduction and abduction motions. The polarization of the CIPS tuned the output current of the CIPS/GaN FeHEMT using the enhancement pulse at its gate node. This nonvolatile artificial synaptic device was connected to the CMOS-based efferent system that integrates and fires the spike to actuate the mechanical platform, such as a mechanical object tracker. The temporal dynamics of the CIPS/GaN FeHEMT were modulated by the enhancement process, analogous to the biological stimulus response. We believe that artificial NMJs with high-power and high-frequency electronic components have the potential to realize functional bioinspired elements for artificial muscles and smart robotic applications.

## MATERIALS AND METHODS

### Device fabrication

Epilayers for a HEMT structure were grown by metal-organic chemical vapor deposition (MOCVD) on a 2-inch silicon substrate (MSE supplies, USA). The heterostructure was etched to isolate the mesa structure via a BCl_3_/Cl_2_-based inductively coupled plasma reactive ion etching (ICP-RIE) system. A Ti/Al (25/140 nm) metal stack was deposited via sputtering, followed by the deposition of Ni/Au (40/50 nm) via an electron-beam (e-beam) evaporator. The sample was annealed using a rapid thermal annealing system at 830°C for 30 s in an N_2_ atmosphere to form ohmic contacts. A 5-nm Al_2_O_3_ gate dielectric was deposited using an atomic layer deposition system with trimethylaluminum and ozone precursors at 450°C. A CIPS flake (60 nm) was then exfoliated from the CIPS bulk (HQ graphene) and transferred to the HEMT channel interface. The final Ni/Au (40/80 nm) gate electrode was deposited by e-beam evaporation.

### Material characterizations

A cross-sectional CIPS/GaN FeHEMT structure was analyzed using a focused ion beam system and high-resolution TEM [JEM-2100F(HR), JEOL Ltd.] with EDS elemental mapping. The PFM amplitude and phase were characterized using a PFM tip with a writing bias range of ±10 V in the electrostatic force microscopy mode (NX-10/Park Systems). Micro-Raman spectroscopy was performed at room temperature using a Raman imaging microscope (DXR 2xi) with a laser excitation wavelength of 532 nm.

### Electrical characterizations

Electrical measurements were performed using a semiconductor parameter analyzer (Keithley 4200A-SCS) equipped with a PMU-4225 (connected to an RPM module). Neuromorphic characterizations were performed using a semiconductor analyzer (B1500A), pulse generator (Keysight 33600A), and oscilloscope (Keysight DSO-X 3024 T). The IFU consisted of a 100-kilohm resistor, 66-nF capacitor, operational amplifier (OPA227), and comparator (LM*_x_*93-N). The pulses were generated and read using a Digilent Analog Discovery 2.

### TCAD simulations

An experimentally measured transfer curve of the CIPS/GaN FeHEMT was fitted using the TCAD simulation tool (Synopsys Company) using the ferroelectric and mobility models (*60*, *61*)

$$P_{\mathrm{aux}}=c\cdot P_{s}\cdot \tanh\left[\frac{1}{2F_{c}}\ln\left(\frac{P_{s}+P_{r}}{P_{s}-P_{r}}\right)\cdot (F_{\mathrm{aux}}\pm F_{c})\right]+P_{\mathrm{off}}$$ (2)

$$\frac{d}{dt}P(t)=\frac{P_{\mathrm{aux}}[F_{\mathrm{aux}}(t)]-p(t)}{\tau_{P}}(1+k_{n}|\frac{d}{dt}F_{\mathrm{aux}}(t)|)$$ (3)

where *P*_aux_ and *F*_aux_ represent the auxiliary polarization and electric field, respectively, calculated from the Poisson equation, and *c* and *P*_off_ reflect the hysteretic characteristics of the material in Eq. 2. *P*_s_ is the saturation polarization (10 × 10^−6^ C/cm^2^), *P*_r_ is the remanent polarization (8 × 10^−6^ C/cm^2^), and *F*_c_ is the coercive field (5 × 10^5^ V/cm) achieved by using the CIPS MFM capacitor. Equation 3 is the actual polarization field [*P*(*t*)] computed in the TCAD simulation, where τ*_P_* and *k_n_* are material-specific constants. We computed *P*(*t*) and the field induced from the CIPS layer using a transient solver. The simulation curve was fitted to the measured data by modifying the variable τ*_P_* in Eq. 3. The mobility models used to calculate the *I*-*V* characteristics were based on the Arora-doping dependence model and transferred electron model. Default GaN parameters were used in the mobility models (*62*).

### In situ object tracking experiment

We used the chaotic Mackey-Glass time-series data for a moving signal, *x*(*t*), generated by the discrete equation (*63*–*66*)

$$x(t+1)=c\cdot x(t)+\left[\frac{a\cdot x(t-d)}{b\cdot x{\left(t-d\right)}^{e}}\right]$$ (4)

where *a* (0.05), *b* (0.2), *c* (0.9), *d* (17), and *e* (10) are constants. The first 100 data points were removed to achieve a stable oscillating chaotic signal. The remaining 400 data points were then normalized between 0 and 1. In this experiment, the output of the ultrasound distance sensor was between 0 and 0.8 V, but further distance ranging is possible when the larger space is available, reaching the operating voltage of the ultrasound distance sensor (5.5 V). Thus, within the maximum possible voltage range of the sensor (5.5 V), we additionally amplified the output signal (59-179, Edmund) that reaches up to 4 V only for the visualization purpose (laser beam movement) as recorded in movie S1. The *I*_DS_ output signals were interpolated from 200 to 400 data points and normalized to match the dimensions with that of *x*(*t*). The tracking spot was illuminated by a 532-nm diode laser (DJ532, Thorlabs).

### MNIST classification simulations

We implemented the MNIST classification ANN architecture using the NeuroSim simulator (*67*). A total of 10,000 training images and 100 test images were used, and the original 28 × 28 MNIST images were resized to 16 × 16. The ANN architecture included a 256-10 single hidden layer with a learning rate of 0.01. The weights in the ANN architecture were programmed on the basis of the LTP of the CIPS/GaN FeHEMT, as shown in fig. S7. The nonlinearity of the LTP curves was mathematically fitted using MATLAB, and the extracted nonlinearity parameters were used in the NeuroSim simulator.
