# Supplementary material for: An artificial neuromuscular junction for enhanced reflexes and oculomotor dynamics based on a ferroelectric CuInP2S6/GaN HEMT
Source: Sci Adv. 2023 Sep 22;9(38):eadh9889. doi: 10.1126/sciadv.adh9889 (PMC10516496; doi:10.1126/sciadv.adh9889)
Supplement: Supplementary file 1 — Figs. S1 to S10 Table S1 Legends for movies S1 and S2 [file sciadv.adh9889_sm.pdf]

Science Advances
AAAS

Supplementary Materials for

An artificial neuromuscular junction for enhanced reflexes and oculomotor dynamics based on a ferroelectric $CuInP_2S_6$/GaN HEMT

Minseong Park *et al.*

Corresponding author: Geonwook Yoo, gwyoo@ssu.ac.kr; Kyusang Lee, kl6ut@virginia.edu

Sci. Adv. **9**, eadh9889 (2023)
DOI: 10.1126/sciadv.adh9889

The PDF file includes:

Figs. S1 to S10
Table S1
Legends for movies S1 and S2

Other Supplementary Material for this manuscript includes the following:

Movies S1 and S2

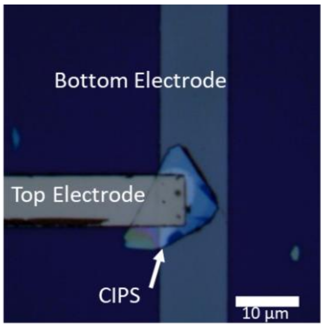

Supplementary figure. S1 Optical microscopy (OM) image of metal-insulator-metal (MIM) $CuInP_2S_6$ (CIPS) structure. The CIPS flake was mechanically exfoliated and transferred to the bottom electrode, followed by the top electrode deposition.

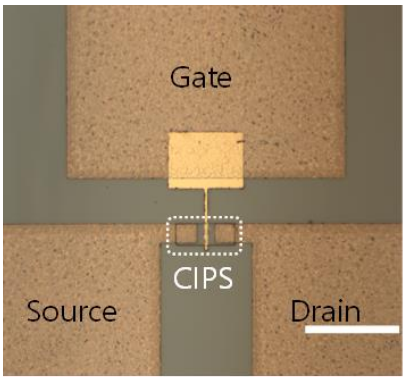

Supplementary figure. S2 OM image of CIPS high-electron-mobility transistor (HEMT).

Scalebar: 50 µm.

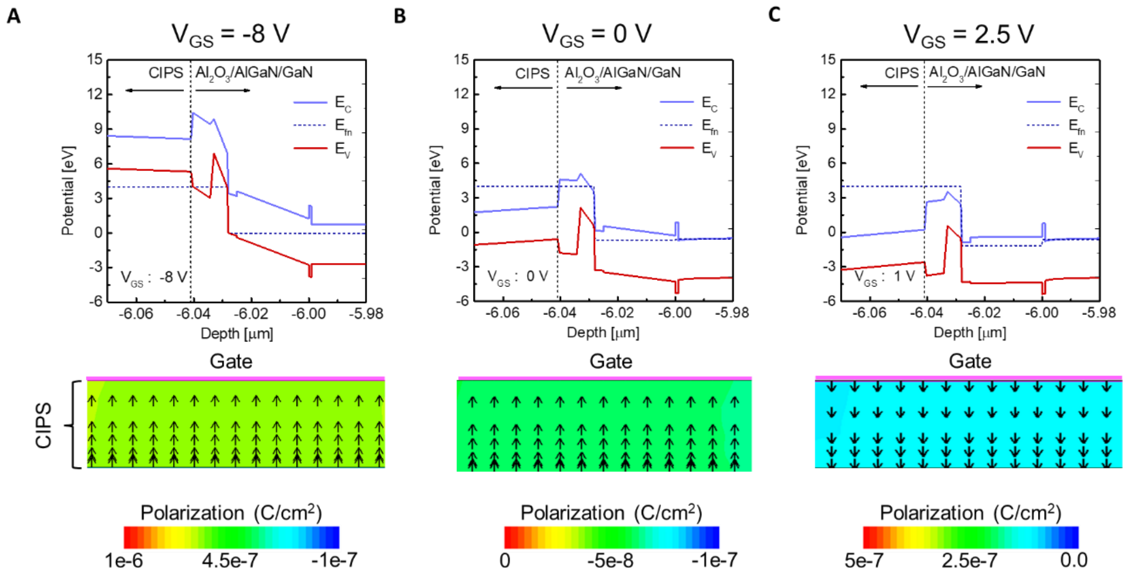

Supplementary figure. S3 Bias-dependent band diagram simulation (TCAD). The barrier height at the CIPS/Al_2O_3 interface is tunable based on the V_{GS}, imparting the reconfigurability to the two-dimensional electron gas (2DEG) channel.

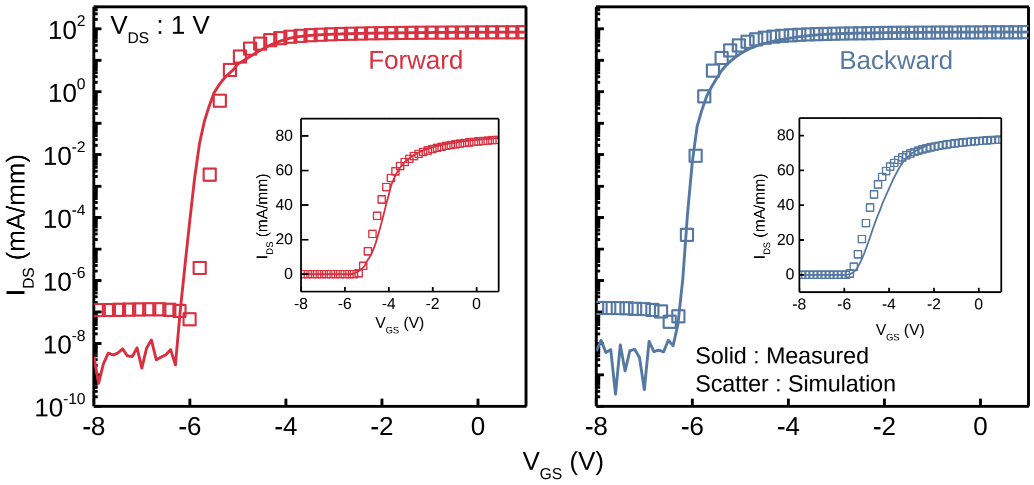

Supplementary figure. S4 Simulated current-voltage (*I*-*V*) characteristics of CIPS HEMT. Experimental transfer curves (lines) of forward (left) and backward (right) with simulated results (dots).

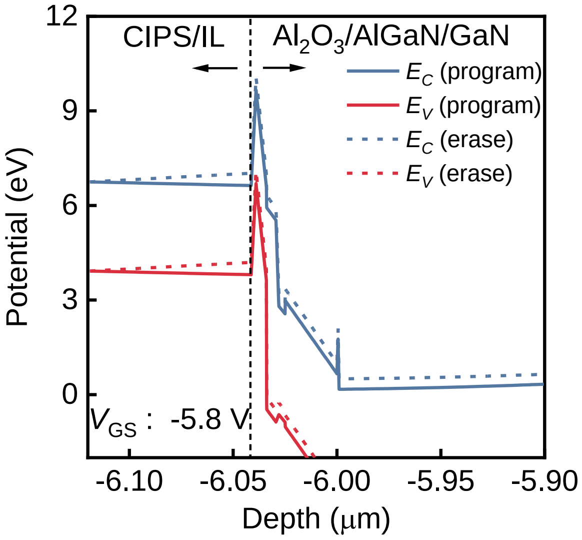

Supplementary figure. S5 Simulated energy band diagram. The barrier height is tunable by polarization states of the CIPS membrane.

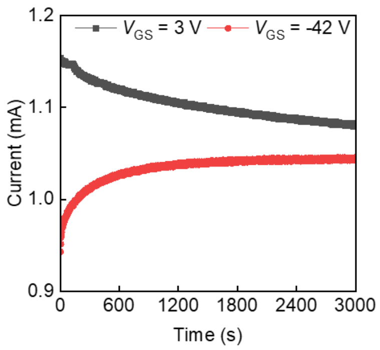

Supplementary figure. S6 Retention time of CIPS/GaN FeHEMT. The pulse width of gate voltage (V_{GS}) was 2 ms in both cases, associated with 2-ms read voltage of -2 V, a baseline of -5 V, and a drain voltage (V_{DS}) of 1 V.

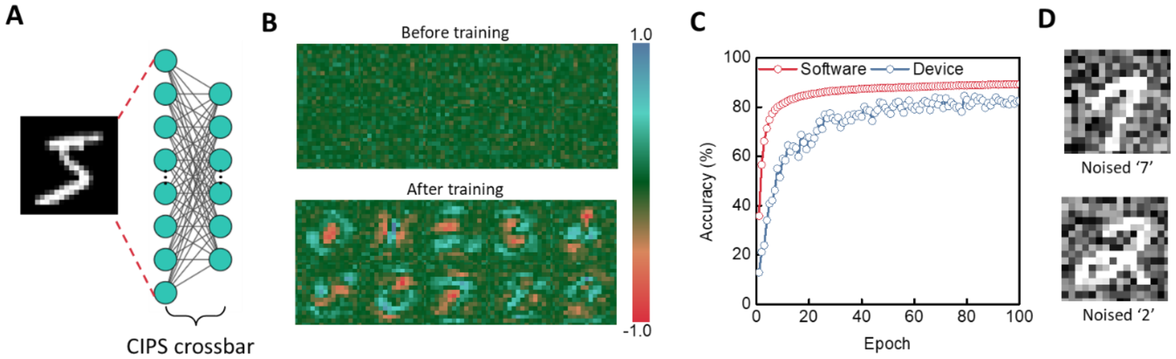

Supplementary figure. S7 Simulation of long-term plasticity (LTP) of CIPS HEMT. (A) Schematic diagram of 256-10 artificial neural network (ANN) with weights representing CIPS crossbar. **(B)** Evolution of weight matrix (256 × 16) reshaped as ten 16 × 16 matrices. **(C)** Results of MNIST classification. The CIPS/GaN ferroelectric crossbar reaches 82% classification accuracy comparable to the pure software result. **(D)** Results of inference of noised '7' and '2' MNIST images. The ANN infers the correct answers for the two noised images, showing the highest probability of the correct labels.

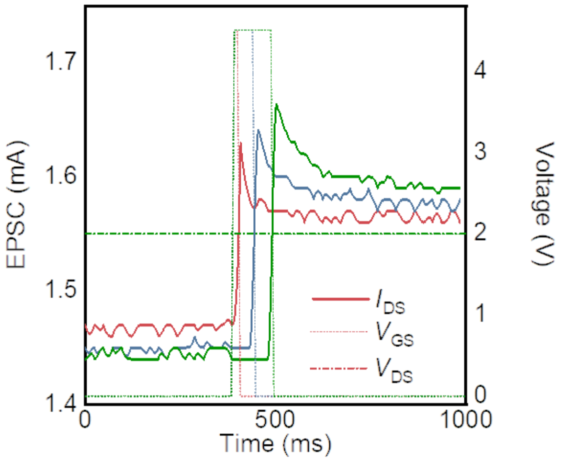

Supplementary figure. S8 Short-term plasticity (STP) of CIPS/GaN FeHEMT. The amplitudes of V_{GS} (program) and V_{DS} (read) are 4.5 V and 2 V, respectively. The pulse widths of V_{GS} includes 10, 50, and 100 ms.

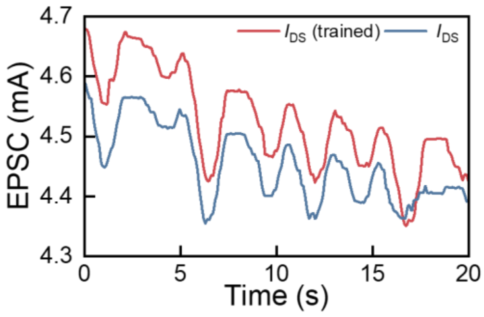

Supplementary figure. S9 Raw data of tracking output time-series data (before normalization). Higher output current level (I_{DS}) more than approximately 4.5 mA in Fig. 4G can also provide high signal-to-noise ratio (SNR), which can be achieved by scaling the width/length (*W*/*L*) ratio of the CIPS/GaN channel for amplifier-less integratability with broader actuator options.

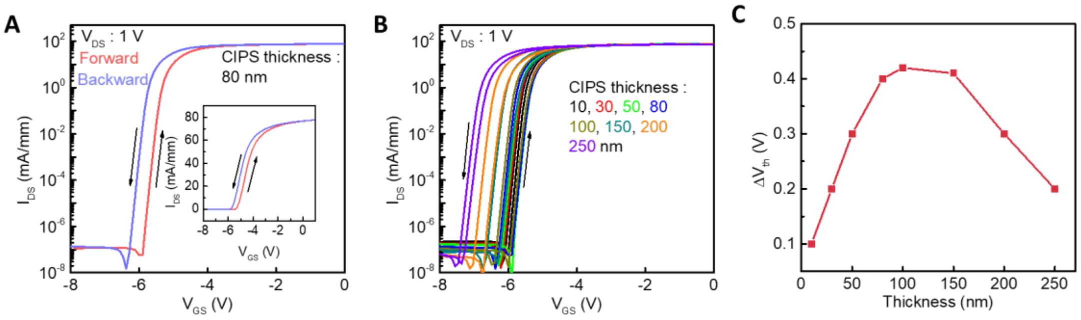

Supplementary figure. S10 Simulated CIPS-thickness-dependent *I-V* characteristics. (A) Round-trip sweep of 80-nm CIPS HEMT. Inset: linear scale plot. **(B)** Round-trip sweeps of 10~250-nm CIPS thicknesses. **(C)** Various ΔV_{th} with respect to various CIPS thicknesses. Among them, 100-nm CIPS leads to the largest ΔV_{th}.

Mechanism	Materials	Device size	Drain current	Synaptic/neuronal functions	Refs.
Ferroelectric	Active layer: AlGaN/GaN<br>Ferroelectric: CIPS	W = 10 μm<br>L = 10 μm	200 mA/mm<br>(@ V_{DS} = 10 V, V_{GS} = 0 V)	LTP, STP, pattern classification	This work
	Active layer: pentacene<br>Ferroelectric: P(VDF-TrFE)	W = 200 μm<br>L = 20 μm	5×10^{-4} mA/mm<br>(@ V_{DS} = -10 V, V_{GS} = -30 V)	LTP, STP, STDP	(42)
	Active layer: MoS_2<br>Ferroelectric: HZO	W = 20 μm<br>L = 3 μm	0.35 mA/mm<br>(@ V_{DS} = 0.1 V, V_{GS} = 5 V)	LTP, pattern recognition/classification	(43)
	Active layer: Si<br>Ferroelectric: HZO	W = 50 μm<br>L = 120 μm	80 mA/mm<br>(@ V_{DS} = 1 V, V_{GS} = 3 V)	LTP	(44)
	Active layer: β-Ga_2O_3<br>Ferroelectric: α-In_2Se_3	W = 10 μm<br>L = 5 μm	0.1 mA/mm<br>(@ V_{DS} = 1 V, V_{GS} = 6 V)	LTP	(45)
Electrolyte	Active layer: PEDOT:Tos/PTHF<br>Electrolyte: NaCl	W = 500 μm<br>L = 10 μm	3.8 mA/mm<br>(@ V_{DS} = -0.2 V, V_{GS} = -0.8 V)	LTP, STP, associative learning	(46)
	Active layer: InZnO<br>Electrolyte: Ion-gel	W = 1600 μm<br>L = 80 nm	0.31 mA/mm<br>(@ V_{DS} = 0.25 V, V_{GS} = 3 V)	LTP, STP, STDP	(47)
	Active layer: NDI-gTVT<br>Electrolyte: PEO/$LiClO_4$	W = 5 μm<br>L = 150 nm	16 mA/mm<br>(@ V_{DS} = -1 V, V_{GS} = 3 V)	LTP, STP, pattern recognition	(48)
Floating-gate	Semiconductor: CNT<br>Floating-gate: Au	W = 3 μm<br>L = 2.6 μm	0.53 mA/mm<br>(@ V_{DS} = 1 V, V_{GS} = -20 V)	LTP, STDP	(49)
	Semiconductor: Pentacene<br>Floating-gate: C_{60}	W = 1000 μm<br>L = 50 μm	0.16 mA/mm<br>(@ V_{DS} = 5 V, V_{GS} = -5 V)	LTP, STP	(50)
Quantum dots	Active layer: $CsPbBr_3$<br>Dielectric: SiO_2	W = 1000 μm<br>L = 50 μm	0.06 mA/mm<br>(@ V_{DS} = -30 V, V_{GS} = -40 V)	LTP, STP	(51)

Supplementary table S1 Comparison of drain current (output current) among recent synaptic transistors. The proposed CIPS/GaN FeHEMT exbhitis high drain current compared to other recent synaptic transistors. LTP: Long-term plasticity. STP: Short-term plasticity. STDP: Spike-timing-dependent plasticity.

Supplementary movie 1 In-situ object tracking. The green last spot tracks the car moving which signal was based on the Mackey-Glass chaotic time series.

Supplementary movie 2 Phase plot of in-situ object tracking. The enhanced device can track the movement closer to the original signal.
